# Prospective observational study of cell-free DNA as a prognostic biomarker in COVID-19 and bacterial sepsis: COVSEP-study

**DOI:** 10.1038/s41598-025-32810-4

**Published:** 2025-12-18

**Authors:** Katharina Hoeter, Elmo W. I. Neuberger, Vanessa Jochum, Robert Kuchen, Kira Enders, Maria Bergmann, Michael K. E. Schäfer, Perikles Simon, Marc Bodenstein

**Affiliations:** 1 Department of Anesthesiology, University Medical Centre of the Johannes Gutenberg-University, Langenbeckstr. 1, 55131 Mainz, Germany; 2 Department of Sports Medicine, Disease Prevention and Rehabilitation, Johannes Gutenberg-University Mainz, Mainz, Germany; 3 Institute of Medical Biostatistics, Epidemiology and Informatics (IMBEI), University Medical Centre of the Johannes Gutenberg-University, Mainz, Germany; 4 Focus Program Translational Neurosciences (FTN), Johannes Gutenberg-University, Mainz, Germany; 5 Research Center for Immunotherapy (FZI), University Medical Centre of the Johannes Gutenberg-University, Mainz, Germany

**Keywords:** Cell-free nucleic acids, Biomarker, Inflammation, COVID-19, Sepsis, Biomarkers, Diseases, Medical research, Microbiology

## Abstract

Hyperinflammation and extensive cell damage characterize both COVID-19-sepsis and bacterial sepsis, contributing to poor clinical outcomes. Cell-free DNA (cfDNA), a damage-associated molecular pattern (DAMP), reflects ongoing tissue injury and may predict mortality. We aimed to evaluate cfDNA as a prognostic biomarker for 30-day mortality in ICU patients with COVID-19- vs. bacterial sepsis, and its association with inflammatory markers and disease progression. In a prospective observational study (ethics approval: 2020–15,535; DRKS-ID: DRKS00025222), cfDNA was quantified in 64 ICU patients (COVID-19-sepsis n = 27, bacterial sepsis n = 37) at four time points using quantitative PCR targeting 90 bp and 222 bp fragments of LINE-1 elements. An Integrity Index (222/90 bp) was calculated to infer the predominant mode of cell death. Nineteen healthy individuals served as controls. Associations with mortality and clinical parameters were analyzed using adjusted Cox regression, time-dependent models, and correlation analyses. Higher cfDNA levels (90 bp) within the first 24 h were strongly associated with 30-day (*p* = 0.003) and 180-day mortality (*p* = 0.003) in COVID-19-sepsis, but not in bacterial sepsis. COVID-19 patients showed significantly higher cfDNA levels (*p* < 0.01), which correlated with CRP, PCT, LDH, and lactate. The Integrity Index increased over time in bacterial sepsis and remained stable in COVID-19-sepsis, but was not predictive of survival. Elevated cfDNA levels were associated with ECMO therapy but not with renal replacement therapy. cfDNA is a valuable early prognostic biomarker in COVID-19-sepsis. Its rapid dynamics and strong correlation with clinical outcomes highlight its potential for real-time monitoring and risk stratification in viral sepsis.

**Supplementary Information:**

The online version contains supplementary material available at 10.1038/s41598-025-32810-4.

## Introduction

Cell-free DNA (cfDNA) is a recognized mediator of inflammation and tissue damage in sepsis syndromes, including both bacterial sepsis and coronavirus disease 2019 (COVID-19) sepsis. Elevated cfDNA levels reflect cellular injury, neutrophil activation, and uncontrolled immune responses, contributing to the amplification of systemic inflammation^1^.

Several conventional biomarkers of inflammation, such as C-reactive protein (CRP), procalcitonin (PCT), D-dimer, and ferritin^2,3^ correlate with adverse outcomes in sepsis. Inflammatory cytokines including interleukin (IL)-6, IL-8, and tumor necrosis factor-α (TNF-α), have also been linked to unfavorable disease trajectories^4–6^. However, cfDNA distinguishes itself as a damage-associated molecular pattern (DAMP), released from dead or damaged cells, or from activated immune cells such as neutrophils^7^ as a consequence of viral or bacterial infection. It plays a distinctive role in actively exacerbating inflammatory responses through pattern recognition receptors (PRRs). This amplification results in a vicious cycle of inflammation and cellular damage, contributing to disease severity^8^. In both COVID-19 and bacterial sepsis, cfDNA promotes the formation of neutrophil extracellular traps (NETs)^9^, a defensive mechanism that, when dysregulated, exacerbates organ injury and inflammation^10^ by releasing additional DAMPs and promoting cell death^8^. In COVID-19 sepsis, these processes can culminate in cytokine release syndrome (CRS) and multi-organ failure (MOF)^11^, mechanisms closely paralleling bacterial sepsis^8^.

Recent studies have identified cfDNA as a predictive biomarker for disease severity in both COVID-19 and bacterial sepsis^12,13^, based on its ability to reflect systemic inflammation, and tissue damage^14^. To further characterize the source and nature of cfDNA, the cfDNA Integrity Index—calculated as the ratio of longer (222 bp) to shorter (90 bp) fragments—has been proposed as an indicator of cfDNA origin. A higher Integrity Index is associated with NETosis and necrotic cell death, as seen in neutrophil activation, while a lower Integrity Index is typically reflective of apoptosis^15^. Importantly, the Integrity Index is not static and may change over time during the course of critical illness, potentially reflecting shifts in the dominant mechanisms of cell death and immune activation.

However, comparisons across studies are limited by heterogeneity in measurement techniques and clinical protocols. Consequently, the prognostic value of cfDNA in viral sepsis versus bacterial sepsis remains uncertain in the absence of a standardized, comparable study designs.

The primary endpoint of this study was to systematically evaluate whether cfDNA levels serve as a prognostic biomarker for 30-day mortality in patients with COVID-19-sepsis, and to compare this association to patients with bacterial sepsis. Additionally, we monitored cfDNA progression during the ICU stay, correlated cfDNA with inflammatory markers and clinical complications, and performed follow-up of patient mortality up to 180 days after ICU admission.

We hypothesized that cfDNA levels would be significantly higher in COVID-19-sepsis patients compared to bacterial sepsis, based on findings from our preceding pilot study, which demonstrated elevated cfDNA levels in COVID-19 patients compared to healthy controls^12^. Furthermore, we anticipated that cfDNA dynamics would correlate with short- and long-term mortality, as well as markers of disease progression, especially given the absence of specific therapeutic interventions for COVID-19-sepsis.

## Methods

### Study description

This prospective observational study was conducted at the Department of Anesthesiology, University Medical Center Mainz, Germany. Ethical approval (Landesärztekammer Rheinland-Pfalz; 2020–15,535) was obtained, following the Declaration of Helsinki and registered in the German Clinical Trial Register (DRKS-ID: DRKS00025222). The study recruited patients in the anesthesiologic ICU from February 2021 to May 2022. Additionally, a control group of 19 healthy individuals, generated in a preceding pilot study (Ethical approval No.: 2020–15,116-retrospective), was included to provide baseline cfDNA reference values.

### Data collection

Patient data and test results were pseudonymized and collected from patients meeting inclusion criteria (Supplementary Table 1). Inclusion criteria were: diagnosis of bacterial or COVID-19 sepsis according to Sepsis-3 definitions^16^. Exclusion criteria comprised pre-existing conditions known to elevate cfDNA levels independently of infection, such as active malignancy or pregnancy. SARS-CoV-2 infection was confirmed by polymerase chain reaction (PCR) test. Patients were followed for 180 days post-ICU admission, with mortality assessed at 30 and 180 days.

Patients with COVID-19 received standardized treatment according to national recommendations of the RKI (Robert Koch Institut) and its STAKOB/COVRIIN group, including Remdesivir and corticosteroids. No deviations such as the interim use of acetylsalicylic acid, ACE inhibitors, or monoclonal antibody therapy occurred.

### Power analysis and study size

Power analysis based on a pilot study^12^ indicated a cohort size of 60 patients per group. The calculation was based on cfDNA levels observed in moderately ill COVID-19 patients and healthy controls. In the current study, patients were more severely ill and showed substantially higher cfDNA levels, which exceeded pilot estimates. In the COVID-19 sepsis cohort, recruitment became increasingly difficult, and univariable Cox proportional hazards models had already demonstrated a highly significant association between cfDNA levels and 30- and 180-day mortality (*p* < 0.001); therefore, only 27 of the planned 60 patients were included. In the bacterial sepsis cohort, recruitment was stopped after 37 patients, as a sample size calculation based on these data indicated that at least 141 patients would be required to detect a significant association between cfDNA levels and mortality, at a significance level of 0.05 and a power of 80%, which was not feasible. Patient enrollment was thus terminated early due to declining ICU admissions and the robustness of the findings.

### Sample collection and analysis

Blood samples were drawn into ethylenediaminetetraacetate (EDTA) tubes (Sarstedt®) at four defined time points: at ICU admission (0–24 h), between days 1–3, between days 7–10, and at day 14 (Supplementary Table 2). Samples were centrifuged at 3746 × g for 10 min at 20 °C within 3 h and stored at -80 °C. Quantitative real-time qPCR was performed for cfDNA determination as described by Neuberger et al.^17^. Amplicons of 90 and 222 base pairs (bp) of human long nuclear elements (LINE) of the L1PA2 family^18^ were analyzed, achieving ultra-low limit of quantification (LOQ) of 0.47 and 0.69 ng/mL, with repeatability ≤ 11.6% and intermediate precision ≤ 12.2%^17^. The cfDNA Integrity Index was calculated as the ratio of the concentration of 222 bp fragments to 90 bp fragments, reflecting the relative proportion of longer to shorter cfDNA fragments. CfDNA levels were determined without altering patient treatment.

The same blood collection tubes (EDTA, Sarstedt®), centrifugation scheme (3746 × g for 10 min, 20 °C, within 3 h of collection), storage conditions (− 80 °C), and identical qPCR assay (primer sets, reagents, platform, and calibration procedures) were used for both patients and controls. This approach minimized potential batch effects and ensured comparability between groups. The decision to use previously enrolled controls was based on ethical reasons, given that recruiting contemporaneous healthy volunteers during the COVID-19 pandemic was restricted.

### Statistical analysis

Statistical analyses were conducted using R software (version 4.2.2, R Foundation for Statistical Computing, Vienna, Austria). Continuous variables are presented as mean ± standard deviation (SD) or median (interquartile range, IQR), and categorical variables as counts and percentages. For regression analyses, cfDNA values were used in their log-transformed form.

#### Primary endpoint

The primary endpoint was the association between cfDNA levels (90 bp and 222 bp), measured within 24 h of ICU admission, and 30-day mortality. This was assessed using Cox regression models adjusted for age and ASA classification. Survival curves were visualized using Kaplan–Meier estimates, with cfDNA levels dichotomized at median.

#### Secondary endpoints

Secondary endpoints focused on evaluating the relationship between cfDNA levels and (1) 180-day mortality, (2) inflammatory biomarkers (e.g., CRP, PCT, LDH, WBC, lactate), and (3) clinical complications such as the need for extracorporeal membrane oxygenation (ECMO) or renal replacement therapy.

1. Associations between cfDNA levels and 180-day mortality were analyzed using Cox regression models, adjusted for age and ASA classification. To further assess the prognostic value of cfDNA over time, extended Cox models with time-dependent covariates were applied.
2. Relationships between cfDNA levels and inflammatory markers at all four sampling time points were examined using Spearman’s rank correlation coefficient. These analyses were performed separately for COVID-19-sepsis and bacterial sepsis groups.
3. Associations between cfDNA concentrations and clinical complications, including the need for organ support therapies such as ECMO or renal replacement therapy, were analyzed using generalized estimating equations (GEE).

Additionally, cfDNA kinetics were evaluated using linear mixed models (ANOVA) to assess group differences and time trends across the four defined sampling points.

Comparison with healthy controls:

cfDNA concentrations and selected laboratory parameters were compared across COVID-19-sepsis, bacterial sepsis, and healthy control groups using the Kruskal–Wallis test.

Significant group differences were followed by Dunn’s post hoc test with Bonferroni correction for pairwise comparisons.

To ensure comparability, only cfDNA values from the first patient sampling (0–24 h after ICU admission) were used in comparisons with the single time-point data from healthy controls.

## Results

### Cohort characteristics

**Fig. 1:**
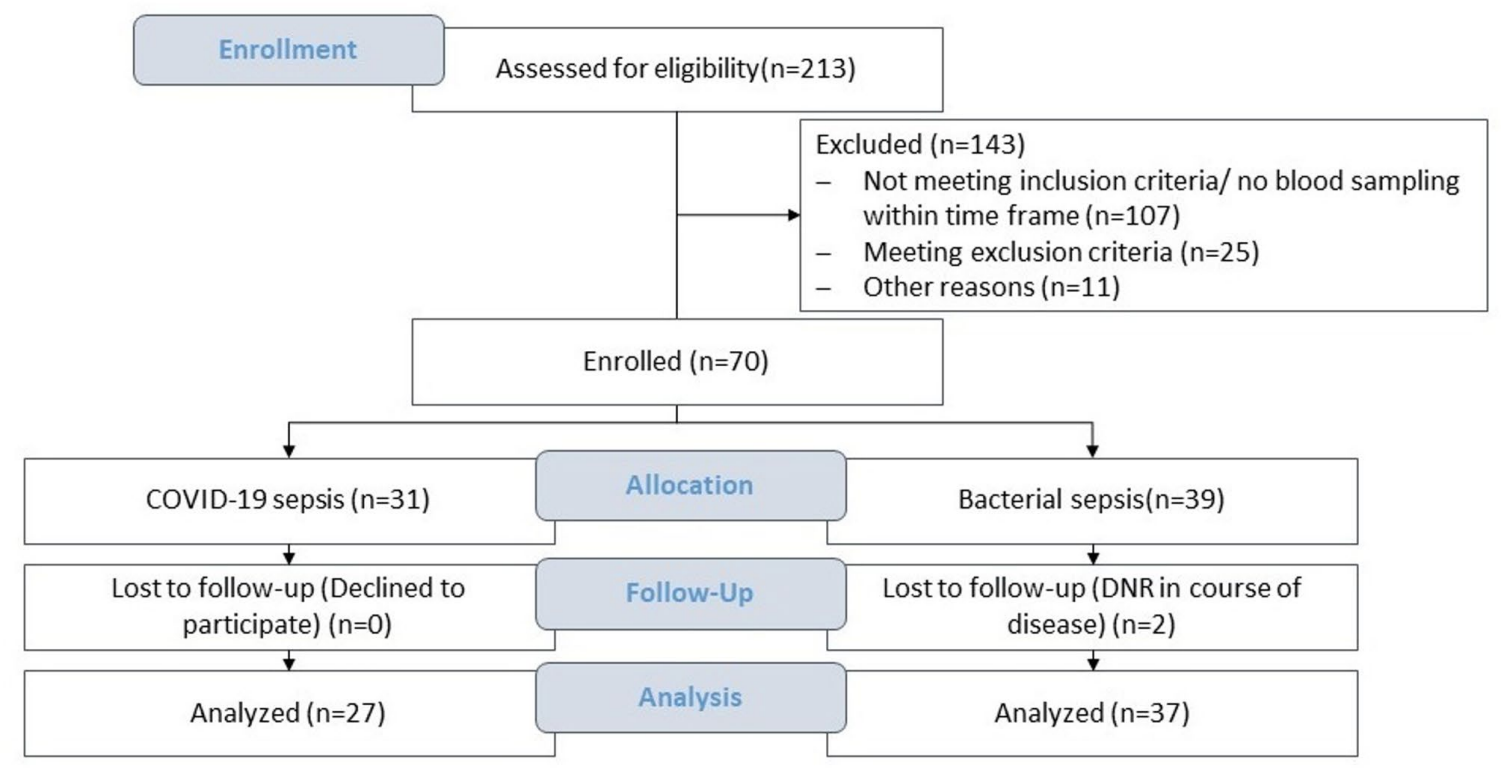
Study cohort flow diagram detailing screening, eligibility, enrollment, and follow-up.

Enrollment included 27 patients with COVID-19-sepsis and 37 patients with bacterial sepsis (Fig. 1, Table 1). Among the COVID-19 patients, the majority were infected with the Delta variant (n = 16, 59.3%), while smaller numbers were observed for Alpha (n = 4, 14.8%), Beta (n = 1, 3.7%), and Omicron (n = 1, 3.7%). In 5 patients (18.5%) the viral variant was not determined. Additionally, a control group of 19 healthy individuals was included for comparison (Supplementary Table 3). Controls had a lower median age compared to patients and exhibited significantly lower cfDNA concentrations at baseline (*p* < 0.001) and significantly higher Integrity Indices (*p* < 0.001). Inflammatory markers such as CRP, WBC, and platelet counts were within normal ranges, with no signs of systemic inflammation.

**Table 1:** Baseline demographic, clinical and laboratory characteristics of ICU patients with COVID-19 sepsis and bacterial sepsis.

Characteristics	COVID-19 sepsis (n = 27)	Bacterial sepsis (n = 37)	Total (n = 64)	*p*-values
Mean age (SD) – yr	54 (19)	72 (12)	64 (18)	< 0.001*^a^
Sex – no. (%)				
Female	9 (33.3)	15 (40.5)	24 (37.5)	0.56
Male	18 (66.7)	22 (59.5)	40 (62.5)	
Pre-existing conditions – no. (%)				
ASA I-II	14 (51.9)	13 (35.1)	27 (42.2)	0.18
ASA III-IV	13 (48.1)	24 (64.2)	37 (57.8)	
Obesity (BMI ≥ 30 kg/m^2^)	15 (55.6)	10 (27)	25 (39.1)	0.02*
Hypertension	14 (51.9)	26 (70.3)	40 (62.5)	0.13
Chronic cardiac disease	4 (14.8)	24 (64.9)	28 (43.8)	< 0.001*
Chronic pulmonary disease	9 (33.3)	13 (35.1)	22 (34.4)	0.88
Chronic renal disease	5 (18.5)	16 (43.2)	21 (32.8)	0.04*
Type 2 diabetes	15 (55.6)	10 (27)	25 (39.1)	0.57
No pre-existing conditions	5 (18.5)	3 (8.1)	8 (12.5)	0.21
Overall cfDNA concentrations arithmetic Mean (SD)				
cfDNA 222 bp (ng/mL)	163 (274)	57 (38)	102 (186)	0.025*^a^
cfDNA 90 bp (ng/mL)	2820 (9967)	429 (530)	1438 (6525)	0.006*^a^
cfDNA concentrations (Median ± IQR) [90 bp (ng/mL)]				
1. Measurement	n = 27 664 (277, 833)	n = 36 234 (160, 604)	n = 64 378 (174, 759)	0.021*
2. Measurement	n = 22 505 (226, 885)	n = 33 226 (136, 343)	n = 55 291 (159, 615)	0.004*
3. Measurement	n = 21 369 (168, 836)	(n = 25) 162 (103, 283)	n = 46 217 (133, 390)	0.005*
4. Measurement	n = 9 297 (131, 454)	n = 17 128 (75, 245)	n = 26 139 (87, 369)	0.26
cfDNA concentrations (Median ± IQR) [222 bp (ng/mL)]				
1. Measurement	n = 27 73 (41, 125)	n = 36 38 (28, 67)	n = 64 52 (30, 89)	0.021*
2. Measurement	n = 22 69 (36, 197)	n = 33 39 (26, 55)	n = 55 44 (34, 80)	0.003*
3. Measurement	n = 21 55 (30, 116)	n = 25 42 (25, 66)	n = 46 46 (26, 85)	0.24
4. Measurement	n = 9 40 (24, 94)	n = 17 29 (22, 50)	n = 26 31 (21, 71)	0.60
Integrity-Index (Mean ± SD) [222 bp (ng/ml): 90 bp (ng/mL)]				
1. Measurement	0.15 (0.07)	0.19 (0.15)	0.17 (0.12)	0.48^a^
2. Measurement	0.18 (0.06)	0.19 (0.11)	0.19 (0.09)	0.92^a^
3. Measurement	0.16 (0.06)	0.31 (0.22)	0.24 (0.18)	< 0.001*^a^
4. Measurement	0.2 (0.12)	0.28 (0.21)	0.25 (0.18)	0.44^a^
Infection parameters (Mean ± SD)				
CRP (mg/L)	135 (99)	236 (98)	194 (109)	< 0.001*^a^
PCT (ng/mL)	1.2 (1.9)	21 (32)	12 (26)	< 0.001*^a^
LDH (U/l)	678 (847)	346 (331)	486 (621)	< 0.001*^a^
Lactate (mmol/L)	2.1 (1.5)	3.2 (3.3)	2.7 (2.7)	0.18^a^
Clinical Outcome Parameters (Mean ± SD)				
SOFA-Score max	13 (5.2)	13 (4.5)	13 (4.8)	0.94^a^
SOFA-Score min	6 (4.3)	5 (4.5)	5.4 (4.4)	0.33^a^
Hospital LOS (d)	25 (25)	38 (26)	32 (27)	0.002*^a^
ICU LOS (d)	18 (20)	16 (18)	17 (19)	0.68^a^
30-day mortality	10 (40%)	11 (31%)	21 (34%)	0.46
180-day mortality	14 (58%)	18 (55%)	32 (56%)	0.78
Complications – no. (%)				
Pulmonary				
ARDS	13 (48)	6 (16)	19 (30)	0.006*
ECMO therapy	7 (26)	1 (3)	8 (12)	0.006*
Duration of ventilation (d)	16 (20)	9.7 (10)	12 (16)	0.047*^a^
Re-Intubation	4 (15)	16 (43)	20 (31)	0.015*
Tracheotomy	9 (33)	4 (11)	13 (20)	0.027*
Minimal Horovitz-Index (Mean ± SD)	81 (38)	139 (74)	114 (67)	< 0.001*
Thromboembolism				
Mesenteric ischemia	0 (0)	3 (8)	3 (5)	0.13
Pulmonary artery embolus	2 (7)	2 (5)	4 (6)	0.75
Neurological				
Delirium	4 (15)	15 (41)	19 (30)	0.026*
Renal				
AKI w/o CRRT	4 (15)	13 (35)	17 (27)	0.07
AKI with CRRT	11 (41)	14 (38)	25 (39)	0.82
Cardiac				
CPR	4 (15)	2 (5)	6 (9)	0.2
Atrial fibrillation	4 (15)	15 (41)	19 (30)	0.026*

*AKI* Acute kidney injury, *ARDS* Acute Respiratory Distress Syndrome, *ASA* American Society of Anesthesiologists Score, *bp* base pairs, *BMI* Body Mass Index, *cfDNA* cell free DNA, *CRP* C-reactive Protein, *CRRT* continuous renal replacement therapy, *ct-value* crossing threshold value, *d,* days, *ECMO* Extra Corporeal Membrane Oxygenation, *IQR* interquartile range*, L* liter, *LDH* Lactate dehydrogenase, *LOS* length of stay, *max* maximum, *mg* milligram, *min* minimum, *mL* milliliter, *mmol* millimoles, *ng* nanogram, * *p* < 0.05, *PCT* Procalcitonin, *SD* standard deviation, *SOFA-Score* Sequential Organ Failure Assessment Score, *U* Units. Pearson’s Chi-squared test unless ^a^ Mann–Whitney’s U test.

Patients with COVID-19-sepsis had similar 30-day (40% vs. 31%, *p* = 0.46) and 180-day mortality (58% vs. 55%, *p* = 0.78) rates compared to bacterial sepsis, but prolonged hospitalization (*p* = 0.002) and a higher prevalence of obesity (*p* = 0.02). Other comorbidities, including chronic cardiac disease and chronic renal disease, were more common in bacterial sepsis. Sequential Organ Failure Assessment (SOFA) scores were comparable between groups. Pulmonary complications, including ARDS and the need for ECMO therapy, were more frequent in COVID-19-sepsis, whereas other complications such as delirium and atrial fibrillation occurred more often in bacterial sepsis.

### Primary endpoints

#### cfDNA levels as a prognostic marker for 30-day mortality

Despite similar 30-day mortality rates between COVID-19 sepsis (40%) and bacterial sepsis patients (31%) (*p* = 0.46; Table 1), cfDNA concentrations were significantly associated with survival outcomes, as illustrated in the Kaplan–Meier curves in Supplementary Fig. 1A–B. In COVID-19 sepsis patients, adjusted Cox regression analysis demonstrated that higher cfDNA levels within the first 24 h of ICU admission were strongly predictive of 30-day mortality (HR 5.02, 95% CI 1.75–14.43; *p* = 0.003; Table 2).

**Table 2:** Cox regression analysis of log-transformed early 90 bp cfDNA levels measured within the first 24 h of ICU admission and 30-/180-day mortality, adjusted for age and ASA-Score.

	30-day mortality	180-day mortality					
Covariate	HR	95%-CI	*p*-value	HR	95%-CI	*p*-value	
COVID-19 sepsis	log cfDNA (90 bp)	5.02	1.75–14.43	0.003*	4.94	1.74–14.01	0.003*
	Age	1.01	0.96–1.07	0.65	1.04	0.99–1.10	0.15
	ASA III-IV vs. I-II	1.29	0.16–10.23	0.81	0.62	0.09–4.49	0.63
Bacterial sepsis	log cfDNA (90 bp)	1.48	0.73–3.02	0.27	1.01	0.56–1.83	0.96
	Age	1.13	1.04–1.21	0.002*	1.11	1.04–1.18	0.001*
	ASA III-IV vs. I-II	1.02	0.29–3.64	0.97	1.06	0.34–3.29	0.96

Hazard ratios (HR) with 95% confidence intervals (CI) are shown; significant results (*p* < 0.05) are marked with an asterisk. *ASA* classification of the American Society of Anesthesiologists, *bp* base pairs.

In contrast, in bacterial sepsis, cfDNA levels did not significantly correlate with 30-day mortality; only patient age was an independent predictor (HR 1.13, 95% CI 1.04–1.21; *p* = 0.002).

ROC analysis demonstrated promising discriminatory ability between survivors and non-survivors at hospital discharge among patients with COVID-19 sepsis (AUC = 0.99), but only moderate discrimination among those with bacterial sepsis (AUC = 0.68).

### Secondary endpoints

#### cfDNA levels and 180-day mortality

Higher cfDNA levels measured within the first 24 h of ICU admission were similarly associated with increased 180-day mortality in COVID-19-sepsis patients (*p* = 0.003; Table 2).

Extended Cox models confirmed that elevated cfDNA concentrations strongly predicted long-term mortality (HR 2.66, 95% CI 1.59–4.45; *p* < 0.001), while in bacterial sepsis no significant relationship between cfDNA levels and 180-day mortality was observed (Supplementary Table 4).

In bacterial sepsis, higher LDH concentrations (*p* = 0.008) and lower WBC counts (*p* = 0.05) were associated with 30-day mortality.

#### cfDNA levels and disease progression over time and correlation with mortality

**Fig. 2:**
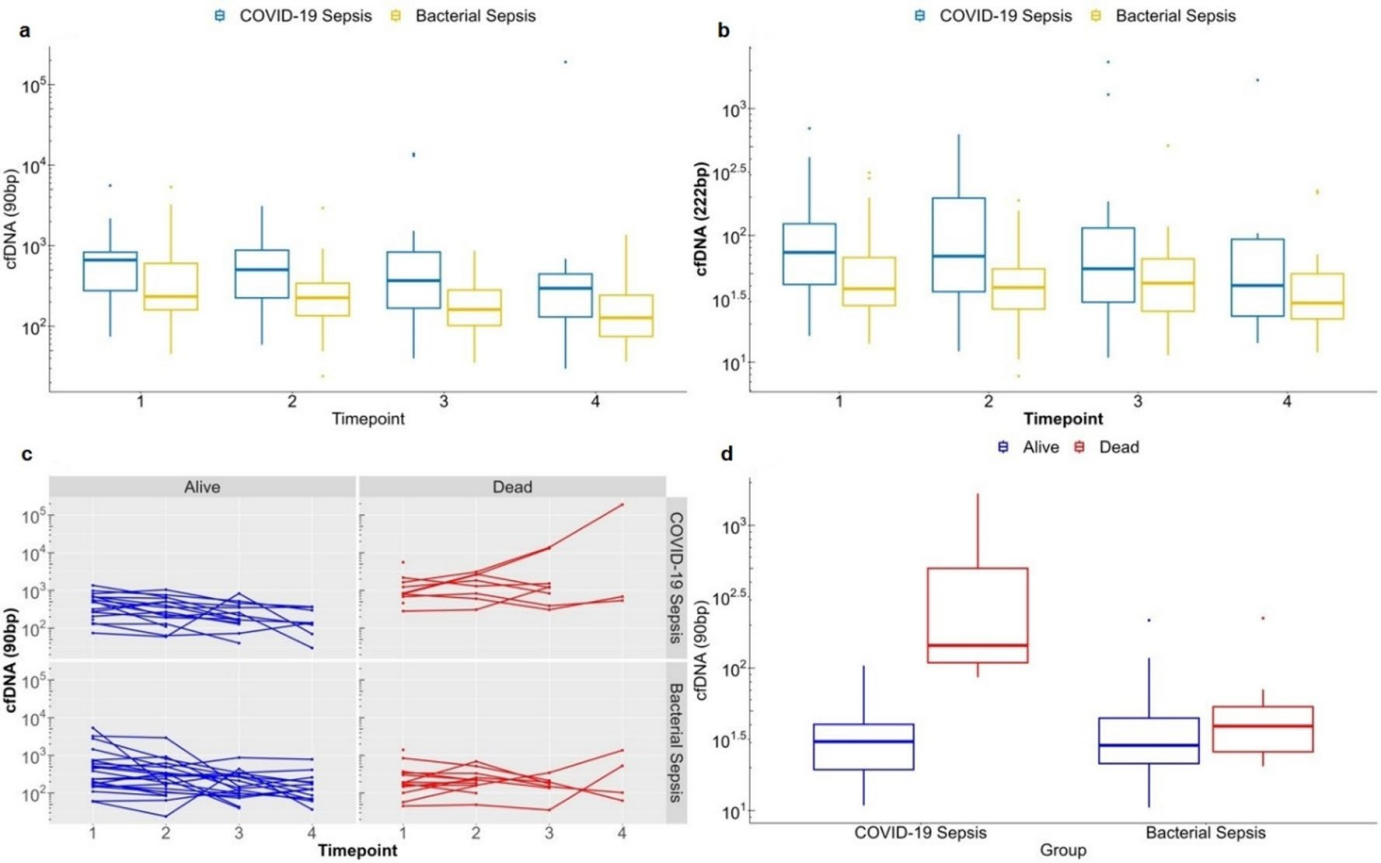
Temporal dynamics and outcome association of log-transformed cfDNA levels in COVID-19 and bacterial sepsis. (**a**) 90 bp cfDNA progression over time for COVID-19-sepsis and bacterial sepsis patients. (**b**) 222 bp cfDNA progression over time for COVID-19-sepsis and bacterial sepsis patients. (**c**) individual courses of cfDNA levels for 90 bp for patients with COVID-19-sepsis and bacterial sepsis separated in survivors and non-survivors. (**d**) cfDNA levels of last measurement and association with hospital discharge and mortality for patients with COVID-19- sepsis and bacterial sepsis. Measurement timepoints:1 = 0–24 h after ICU admission; 2 = day 1–3; 3 = day 7–10; 4 = day 14.

cfDNA levels at ICU admission were significantly higher in COVID-19-sepsis compared to bacterial sepsis for both 90 bp (*p* = 0.001) and 222 bp (*p* = 0.002) fragments (Fig. 2A–B). Over the course of ICU stay, 90 bp cfDNA levels decreased significantly in both groups (*p* = 0.006), while 222 bp levels remained relatively stable (*p* = 0.31).

In COVID-19-sepsis non-survivors showed consistently higher cfDNA levels over time than survivors, with diverging trajectories emerging early during the ICU course (Fig. 2C). This pattern was also visible at the final time point (day 14), where boxplots illustrate substantial differences in cfDNA distributions between survivors and non-survivors (Fig. 2D).

To assess the prognostic relevance of cfDNA kinetics, extended Cox regression models with time-dependent covariates were applied. These models account for dynamic changes in cfDNA levels over time rather than relying on static baseline values. In COVID-19-sepsis, cfDNA progression was independently associated with 30-day mortality (HR 1.45, 95% CI 1.12–1.87; *p* = 0.005; Supplementary Table 4), highlighting its value as a dynamic prognostic biomarker. In contrast, no such association was observed in bacterial sepsis.

The cfDNA Integrity Index (calculated as the ratio of 222 bp to 90 bp fragments) was not significantly associated with survival in either cohort.

#### Correlation of cfDNA with inflammatory markers

cfDNA levels correlated strongly with inflammatory and infection parameters at multiple time points (Supplementary Tables 5 and 6).

In COVID-19-sepsis, the highest correlation was found between cfDNA and LDH at baseline (Spearman’s Rho = 0.79; *p* < 0.0001) and persisted over time. Additionally a significant correlation was observed with CRP (*p* = 0.015) and PCT (*p* = 0.009).

In bacterial sepsis, cfDNA correlated significantly with LDH (*p* < 0.001) and PCT (*p* = 0.02) at baseline, and with lactate from the second time point onward.

#### Influence of pre-existing conditions on cfDNA levels

In COVID-19-sepsis, patients with pre-existing kidney disease (n = 5) exhibited significantly higher cfDNA levels compared to those without (*p* = 0.008) (Supplementary Fig. 2).

Importantly, patients with bacterial sepsis and pre-existing immunological disorders (n = 4) – including autoimmune diseases (systemic lupus erythematosus, rheumatoid arthritis) and heparin-induced thrombocytopenia (HIT) – exhibited significantly elevated baseline cfDNA levels (*p* < 0.001) (Supplementary Fig. 3).

To account for the overall comorbidity burden, outcome analyses were adjusted for ASA classification rather than for individual diseases, as ASA reflects a cumulative comorbidity risk. In these adjusted models, ASA classification itself was not significantly associated with mortality, whereas cfDNA levels in COVID-19-sepsis remained predictive (Table 2).

#### cfDNA and clinical complications

cfDNA concentrations were not significantly associated with acute kidney injury (AKI) or the need for renal replacement therapy (RRT).

However, ECMO therapy was significantly associated with higher cfDNA levels (*p* < 0.001) (Supplementary Table 7).

## Discussion

This study contributes to the growing recognition of cfDNA as a critical biomarker in the pathophysiology of sepsis, particularly in distinguishing the inflammatory responses associated with COVID-19-sepsis and bacterial sepsis. While previous studies have reported its prognostic value in both types of sepsis ^12,13^, cross-group comparisons have been limited due to heterogeneous methods and patient cohorts. By applying a standardized study protocol, we evaluated whether cfDNA levels predict 30-day mortality in patients with COVID-19-sepsis and compared this with bacterial sepsis. Our hypothesis was based on findings from our pilot study in COVID-19 patients and cfDNA data from the literature. In addition, we assessed cfDNA kinetics, associations with inflammatory markers, and outcomes up to 180 days. We found that cfDNA levels were significantly elevated in COVID-19-sepsis, correlated strongly with both short- and long-term mortality, and tracked disease progression. No such prognostic association was observed in bacterial sepsis.

### Primary Endpoints

#### cfDNA as a prognostic marker for 30-day and 180-day mortality

This study demonstrates that cfDNA levels within the first 24 h of ICU admission are strong and independent predictors of 30-day and 180-day mortality in patients with COVID-19-sepsis. These findings confirm and extend results from our earlier pilot study^12^, showing that elevated cfDNA levels are associated with poor outcomes in COVID-19^19^. In contrast, cfDNA concentrations in bacterial sepsis showed no significant association with mortality.

While the fundamental pathophysiological mechanisms of sepsis—such as immune activation, cytokine release, and NET-formation—are shared between bacterial and viral etiologies^9,20^, two key factors may explain the observed differences in cfDNA–mortality associations. First, COVID-19 appears to trigger a more sustained and dysregulated immune response, often characterized by excessive NETosis and delayed viral clearance^21^. This may lead to prolonged cfDNA release and amplified tissue damage. Second, the availability of effective and timely antibiotic treatment in bacterial sepsis likely attenuates the inflammatory cascade early in the disease course^16^, reducing cfDNA levels and their prognostic utility. In contrast, pathogen-specific therapies for COVID-19 were either unavailable or ineffective during the study period^22^, resulting in persistently elevated cfDNA in critically ill patients. These differences were observed despite comparable disease severity at ICU admission, as indicated by similar SOFA scores in both patient groups.

This combination—a stronger pathophysiological immune reaction in COVID-19 and the lack of effective early intervention—may help to explain the association between cfDNA and mortality observed in COVID-19-sepsis. Extended Cox models suggested that cfDNA dynamics over time were independently associated with both short- and long-term mortality in COVID-19 patients, but not in those with bacterial sepsis. The promising discrimination observed in ROC analysis (AUC = 0.99) and Kaplan–Meier survival differences underscore its potential for early risk stratification in viral sepsis. However, these results must be interpreted with caution due to small sample size and should be regarded as hypothesis-generating rather than definitive and require external validation before clinical application. In bacterial sepsis, by contrast, the impact of early treatment may obscure the prognostic relevance of cfDNA.

Previous studies showing prognostic associations in bacterial sepsis^13,23–25^ often lacked longitudinal sampling, standardized protocols, or clear exclusion criteria. Our findings emphasize that cfDNA should be interpreted in the context of both immune activation and therapeutic timing, and support its use as a dynamic, disease- and treatment-sensitive biomarker.

### Secondary endpoints

#### cfDNA levels and disease progression over time

The temporal dynamics of cfDNA in our study provide insight into the evolving mechanisms of cell death during sepsis. While cfDNA concentrations were significantly elevated in COVID-19-sepsis compared to bacterial sepsis early after ICU admission, both groups exhibited a notable decline in 90 bp cfDNA over time, while 222 bp levels remained relatively stable in bacterial sepsis.

The Integrity Index is calculated as the ratio of 222 bp to 90 bp fragments. Short cfDNA fragments (~ 90 bp) are typically associated with apoptotic cleavage patterns, whereas longer fragments (~ 222 bp) may rise from necrosis or NETosis^13,26^. Thus, the observed decline of 90 bp levels over time, together with relatively stable 222 bp levels, is compatible with a shift toward NETosis or necrotic cell death in bacterial sepsis^27^.

In COVID-19-sepsis, both 90 bp and 222 bp levels decreased, resulting in a less pronounced rise of the Integrity Index. This may reflect ongoing apoptosis and less consistent NET-driven fragmentation, in line with prior findings of prolonged immune dysregulation and impaired viral clearance in severe COVID-19^19^.

Overall, these kinetic patterns suggest that cfDNA kinetics may provide information about disease severity as well as about qualitative changes in cell death pathways over time.

However, the Integrity Index was not independently predictive in our models, and its mechanistic interpretation remains speculative. Future studies should therefore combine cfDNA fragment analysis with direct markers of NETosis or nucleosome assays to validate and expand these exploratory findings.

#### Correlation with inflammatory markers

cfDNA showed consistent correlations with established inflammatory biomarkers including CRP, PCT, LDH, and lactate. In COVID-19-sepsis, the strongest association was observed between cfDNA and LDH—a marker of cellular damage—highlighting cfDNA’s ability to reflect active tissue injury. These results confirm earlier reports linking cfDNA to systemic inflammation and cell death^24^, and further establish its utility as a biomarker that integrates both immune activation and tissue damage signals.

Importantly, cfDNA appears to offer key advantages over conventional markers. Unlike CRP and PCT, which depend on hepatic synthesis and exhibit delayed kinetics, cfDNA is released immediately upon cellular damage and has a short half-life (5–120 min), allowing for earlier detection of dynamic inflammatory changes^28^. Furthermore, cfDNA is not affected by hepatic dysfunction or protein synthesis disorders, which can limit the reliability of CRP and PCT in critically ill patients. These properties position cfDNA as a potentially superior biomarker for early-phase disease monitoring and treatment response.

#### cfDNA and pre-existing medical conditions

We also observed that certain comorbidities were associated with altered cfDNA levels. In COVID-19-sepsis, patients with chronic kidney disease had significantly higher cfDNA, likely due to impaired renal clearance, consistent with prior studies^29^. In bacterial sepsis, elevated cfDNA was associated with pre-existing immunological disorders, such as autoimmune disease or HIT^30^, suggesting a possible link between immune dysregulation and enhanced cfDNA release. To account for the overall burden of comorbidities, outcome analyses were adjusted for ASA classification rather than individual diseases, since ASA reflects cumulative comorbidity risk. This approach was chosen because of the limited sample size and the low frequency of many single conditions, which would not allow stable statistical adjustment. In these models, ASA classification itself was not significantly associated with mortality, whereas cfDNA levels in COVID-19-sepsis retained prognostic significance. This indicates that the predictive value of cfDNA is not merely explained by comorbidity burden.

While group sizes were limited, these findings highlight the potential of cfDNA as a comorbidity independent prognostic marker and warrant confirmation in larger studies with detailed phenotyping.

#### cfDNA and clinical complications

Among critically ill patients, extracorporeal membrane oxygenation (ECMO) was significantly associated with elevated cfDNA levels. This may be attributed to the mechanical stress and exposure to artificial surfaces inherent to ECMO circuits, which can promote immune activation and cellular injury^31^. While earlier reports suggested only mild effects of ECMO on cfDNA, our findings indicate a more substantial impact in the context of severe viral sepsis. In contrast, no significant associations were observed between cfDNA and renal replacement therapy or dialysis dependence, possibly due to the use of continuous modalities (CRRT) in our ICU, which are associated with lower shear stress compared to intermittent hemodialysis^32^.

## Limitations

This study provides valuable insights into cfDNA as a biomarker in sepsis; however, several limitations must be acknowledged. The healthy control group was substantially younger and was collected during an earlier study period compared to the patient cohorts. This could bias baseline cfDNA comparisons. To our knowledge, no specific age-stratified reference values for cfDNA are available in the literature; however, it is reasonable to assume that older individuals may exhibit higher cfDNA concentrations. Although identical sampling, processing, storage and qPCR quantification procedures were used to minimize batch effects, residual confounding related to sample handling cannot be excluded. Although regression models were adjusted for age and ASA class, residual confounding from baseline comorbidities cannot be excluded. Furthermore, no statistical adjustment was performed for therapies with antibiotics, antivirals, or corticosteroids, which may have influenced cfDNA levels and outcomes. A residual bias from these factors therefore cannot be excluded. The relatively small sample size, particularly in subgroup analyses, may limit statistical power and inflate effect estimates such as the very high AUC values. Moreover, due to the limited sample size, no internal validation procedures (such as bootstrapping or cross-validation) were performed for the ROC analyses; therefore, a potential risk of overfitting cannot be fully excluded and should be considered when interpreting the discriminatory performance of cfDNA. In addition, the single-center design restricts the generalizability of our findings. Furthermore, the predominance of Delta infections among COVID-19 patients in our cohort may limit the transferability of our results to other SARS-CoV-2 variants.

In addition, while cfDNA reflects systemic inflammation and tissue damage, it lacks cell-type specificity. Although the Integrity Index offers indirect insights into cell death mechanisms, more precise methods—such as methylation profiling or fragmentomics would be needed to determine cfDNA tissue origin and improve biological interpretation.

For future clinical translation, standardized, high-frequency cfDNA sampling and real-time quantification protocols are needed. Future studies should include methylated cfDNA analysis and more granular clinical documentation to enable sepsis subtyping and improve the biomarker’s diagnostic and prognostic utility.

## Conclusions

cfDNA is a promising biomarker in sepsis, with significantly elevated levels in COVID-19-sepsis that were strongly associated with 30- and 180-day mortality. No such association was observed in bacterial sepsis, likely due to effective early treatment and different cfDNA kinetics. Fragment analysis revealed a shift from apoptosis to NETosis over time, particularly in bacterial sepsis, as reflected by the rising Integrity Index, however this interpretation requires further validation. cfDNA also correlated well with inflammatory markers and offers advantages over conventional biomarkers due to its rapid kinetics and independence from hepatic function. These findings support cfDNA’s potential for real-time risk stratification and disease monitoring in critically ill patients, especially in viral sepsis. However, the results should be regarded as hypothesis-generating and require confirmation in larger, multi-center studies with standardized sampling protocols and extended molecular analyses.

## Supplementary Information

Supplementary Information 1.

Supplementary Information 2.

Supplementary Information 3.

Supplementary Information 4.

Supplementary Information 5.

Supplementary Information 6.

Supplementary Information 7.

Supplementary Information 8.

Supplementary Information 9.

Supplementary Information 10.

## Data Availability

The data used and/or analyzed during the current study are available from the corresponding author on reasonable request.
