# Supplementary material for: Prospective observational study of cell-free DNA as a prognostic biomarker in COVID-19 and bacterial sepsis: COVSEP-study
Source: Sci Rep. 2025 Dec 18;15:44144. doi: 10.1038/s41598-025-32810-4 (PMC12717081; doi:10.1038/s41598-025-32810-4)
Supplement: Supplementary file 1 — Supplementary Information 1. [file 41598_2025_32810_MOESM1_ESM.docx]

**Prospective observational study of cell-free DNA as a prognostic biomarker in COVID-19 and bacterial sepsis**

**COVSEP-Study**

Katharina Hoeter^1^, Elmo W.I. Neuberger^2^, Vanessa Jochum^1^, Robert Kuchen^3^, Kira Enders^2^, Maria Bergmann^1^, Michael K. E. Schäfer^1,4,5^, Perikles Simon^2^, Marc Bodenstein^1^

^1^Department of Anesthesiology, University Medical Centre of the Johannes Gutenberg-University, Mainz, Ger-many

^2^Department of Sports Medicine, Disease Prevention and Rehabilitation, Johannes Gutenberg-University Mainz, Mainz, Germany

^3^Institute of Medical Biostatistics, Epidemiology and Informatics, University Medical Centre of the Johannes Gutenberg-University, Mainz, Germany

^4^Focus Program Translational Neurosciences (FTN), Johannes Gutenberg-University, Mainz, Germany

^5^Research Center for Immunotherapy, University Medical Centre of the Johannes Gutenberg- University, Mainz, Germany

Corresponding author:

Katharina Hoeter, MD

katharina.hoeter@unimedizin-mainz.de

ORCID: 0000-0003-4392-9672

**Supplementary Figure 1:** Kaplan Meier estimates for survival after 30/180 days, dichotomized for median of 90 bp cfDNA.

**
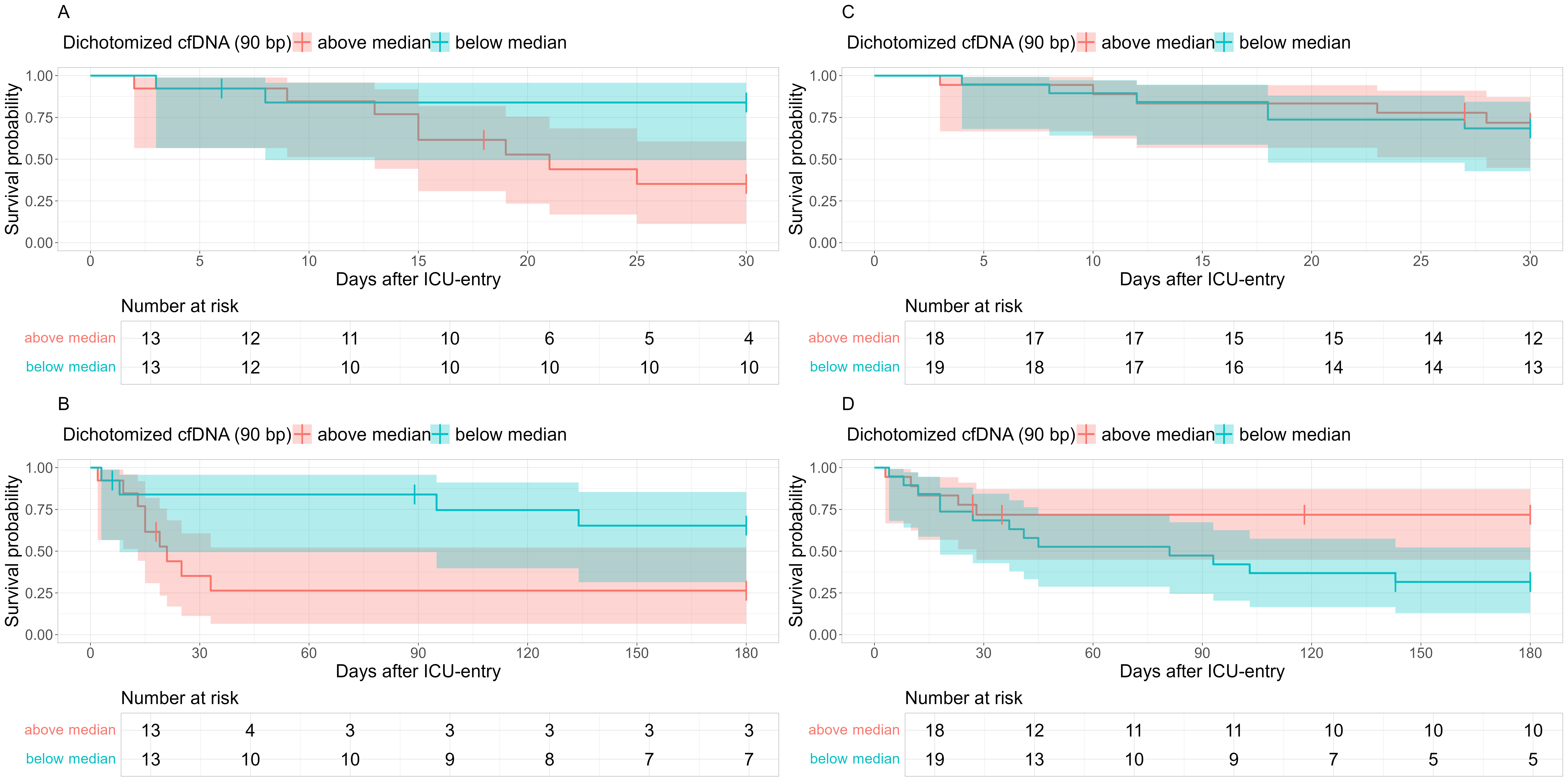
 A** 30-day survival probability in patients with COVID-19-sepsis. **B** 180-day survival probability in patients with COVID-19-sepsis. **C** 30-day survival probability in patients with bacterial sepsis. **D** 180-day survival probability in patients with bacterial sepsis.
