# Supplementary material for: Prospective observational study of cell-free DNA as a prognostic biomarker in COVID-19 and bacterial sepsis: COVSEP-study
Source: Sci Rep. 2025 Dec 18;15:44144. doi: 10.1038/s41598-025-32810-4 (PMC12717081; doi:10.1038/s41598-025-32810-4)
Supplement: Supplementary file 4 — Supplementary Information 4. [file 41598_2025_32810_MOESM4_ESM.docx]

**Prospective observational study of cell-free DNA as a prognostic biomarker in COVID-19 and bacterial sepsis**

**COVSEP-Study**

Katharina Hoeter^1^, Elmo W.I. Neuberger^2^, Vanessa Jochum^1^, Robert Kuchen^3^, Kira Enders^2^, Maria Bergmann^1^, Michael K. E. Schäfer^1,4,5^, Perikles Simon^2^, Marc Bodenstein^1^

^1^Department of Anesthesiology, University Medical Centre of the Johannes Gutenberg-University, Mainz, Ger-many

^2^Department of Sports Medicine, Disease Prevention and Rehabilitation, Johannes Gutenberg-University Mainz, Mainz, Germany

^3^Institute of Medical Biostatistics, Epidemiology and Informatics, University Medical Centre of the Johannes Gutenberg-University, Mainz, Germany

^4^Focus Program Translational Neurosciences (FTN), Johannes Gutenberg-University, Mainz, Germany

^5^Research Center for Immunotherapy, University Medical Centre of the Johannes Gutenberg- University, Mainz, Germany

Corresponding author:

Katharina Hoeter, MD

katharina.hoeter@unimedizin-mainz.de

ORCID: 0000-0003-4392-9672

**Supplementary Table 1**: Inclusion and exclusion criteria for study population enrollment

| Inclusion Criteria | Exclusion Criteria |
| --- | --- |
| ≥ 18 years of age | Pregnancy |
| Acute SARS-CoV-2-infection with sepsis syndrome | Breastfeeding women |
| Bacterial sepsis according to Sepsis-3definitions without prior SARS-CoV-2-infection | Known active tumor disease |
|  | DNR order  DNI order  DNARRT order |
|  | Inclusion stop at escalation level 4 according to SOP of COVID supply |

*DNARRT* Do Not Apply Renal Replacement Therapy, *DNI* Do Not Intubate, *DNR* Do Not Resuscitate, *SOP* Standard Operating Procedure
