# Supplementary material for: Prospective observational study of cell-free DNA as a prognostic biomarker in COVID-19 and bacterial sepsis: COVSEP-study
Source: Sci Rep. 2025 Dec 18;15:44144. doi: 10.1038/s41598-025-32810-4 (PMC12717081; doi:10.1038/s41598-025-32810-4)
Supplement: Supplementary file 5 — Supplementary Information 5. [file 41598_2025_32810_MOESM5_ESM.docx]

**Prospective observational study of cell-free DNA as a prognostic biomarker in COVID-19 and bacterial sepsis**

**COVSEP-Study**

Katharina Hoeter^1^, Elmo W.I. Neuberger^2^, Vanessa Jochum^1^, Robert Kuchen^3^, Kira Enders^2^, Maria Bergmann^1^, Michael K. E. Schäfer^1,4,5^, Perikles Simon^2^, Marc Bodenstein^1^

^1^Department of Anesthesiology, University Medical Centre of the Johannes Gutenberg-University, Mainz, Ger-many

^2^Department of Sports Medicine, Disease Prevention and Rehabilitation, Johannes Gutenberg-University Mainz, Mainz, Germany

^3^Institute of Medical Biostatistics, Epidemiology and Informatics, University Medical Centre of the Johannes Gutenberg-University, Mainz, Germany

^4^Focus Program Translational Neurosciences (FTN), Johannes Gutenberg-University, Mainz, Germany

^5^Research Center for Immunotherapy, University Medical Centre of the Johannes Gutenberg- University, Mainz, Germany

Corresponding author:

Katharina Hoeter, MD

katharina.hoeter@unimedizin-mainz.de

ORCID: 0000-0003-4392-9672

**Supplementary Table 2**: Overview of study design and sampling time points

| Parameters | 0 | 1 | 2 | 3 | 4 | 5 |
| --- | --- | --- | --- | --- | --- | --- |
| Time after study enrollment | 0-24h | 1-3d | 7-10d | 14d | 30d | 180d |
| Review of inclusion and exclusion criteria | X |  |  |  |  |  |
| Study enrollment | X |  |  |  |  |  |
| Assessment of standard laboratory parameters | X | X | X | X* |  |  |
| cfDNA quantification | X | X | X | X* |  |  |
| SOFA-Score | X | X | X | X* |  |  |
| Mortality |  |  |  | X | X | X |

*d* day, *SOFA-Score* Sequential Organ Failure Assessment Score, *h* hours, * optional survey, given that the patient is still hospitalized
