# Supplementary material for: Prospective observational study of cell-free DNA as a prognostic biomarker in COVID-19 and bacterial sepsis: COVSEP-study
Source: Sci Rep. 2025 Dec 18;15:44144. doi: 10.1038/s41598-025-32810-4 (PMC12717081; doi:10.1038/s41598-025-32810-4)
Supplement: Supplementary file 6 — Supplementary Information 6. [file 41598_2025_32810_MOESM6_ESM.docx]

**Prospective observational study of cell-free DNA as a prognostic biomarker in COVID-19 and bacterial sepsis**

**COVSEP-Study**

Katharina Hoeter^1^, Elmo W.I. Neuberger^2^, Vanessa Jochum^1^, Robert Kuchen^3^, Kira Enders^2^, Maria Bergmann^1^, Michael K. E. Schäfer^1,4,5^, Perikles Simon^2^, Marc Bodenstein^1^

^1^Department of Anesthesiology, University Medical Centre of the Johannes Gutenberg-University, Mainz, Ger-many

^2^Department of Sports Medicine, Disease Prevention and Rehabilitation, Johannes Gutenberg-University Mainz, Mainz, Germany

^3^Institute of Medical Biostatistics, Epidemiology and Informatics, University Medical Centre of the Johannes Gutenberg-University, Mainz, Germany

^4^Focus Program Translational Neurosciences (FTN), Johannes Gutenberg-University, Mainz, Germany

^5^Research Center for Immunotherapy, University Medical Centre of the Johannes Gutenberg- University, Mainz, Germany

Corresponding author:

Katharina Hoeter, MD

katharina.hoeter@unimedizin-mainz.de

ORCID: 0000-0003-4392-9672

**Supplementary Table 3**: Baseline demographic and laboratory characteristics of healthy controls

| **Parameter** | **N (%) or mean (± SD)** |
| --- | --- |
| Characteristics | |
| Median age | 43 (11.1) |
| Female | 9 (47.4) |
| Male | 10 (52.6) |
| BMI | 23.83 (4.1) |
| cfDNA | |
| cfDNA 90 bp ng/mL | 14.3 (6.1) |
| cfDNA 222 bp ng/mL | 5.64 (2.7) |
| Integrity Index | 0.39 (0.08) |
| Infection parameters | |
| CRP >5 mg/L | 0.83 (0.9) |
| WBC >10/nL | 6.48 (1.4) |
| Platelets <165/nL | 247.05 (58.8) |

*bp* base pairs, *cfDNA* cell free DNA, *l* liter, *mg* milligram, *ml* milliliter, *ng* nanogram, *nl* nanoliter, *SD* standard deviation, *WBC* white blood cells
