# Supplementary material for: Prospective observational study of cell-free DNA as a prognostic biomarker in COVID-19 and bacterial sepsis: COVSEP-study
Source: Sci Rep. 2025 Dec 18;15:44144. doi: 10.1038/s41598-025-32810-4 (PMC12717081; doi:10.1038/s41598-025-32810-4)
Supplement: Supplementary file 7 — Supplementary Information 7. [file 41598_2025_32810_MOESM7_ESM.docx]

**Prospective observational study of cell-free DNA as a prognostic biomarker in COVID-19 and bacterial sepsis**

**COVSEP-Study**

Katharina Hoeter^1^, Elmo W.I. Neuberger^2^, Vanessa Jochum^1^, Robert Kuchen^3^, Kira Enders^2^, Maria Bergmann^1^, Michael K. E. Schäfer^1,4,5^, Perikles Simon^2^, Marc Bodenstein^1^

^1^Department of Anesthesiology, University Medical Centre of the Johannes Gutenberg-University, Mainz, Ger-many

^2^Department of Sports Medicine, Disease Prevention and Rehabilitation, Johannes Gutenberg-University Mainz, Mainz, Germany

^3^Institute of Medical Biostatistics, Epidemiology and Informatics, University Medical Centre of the Johannes Gutenberg-University, Mainz, Germany

^4^Focus Program Translational Neurosciences (FTN), Johannes Gutenberg-University, Mainz, Germany

^5^Research Center for Immunotherapy, University Medical Centre of the Johannes Gutenberg- University, Mainz, Germany

Corresponding author:

Katharina Hoeter, MD

katharina.hoeter@unimedizin-mainz.de

ORCID: 0000-0003-4392-9672

**Supplementary Table 4:** Extended Cox model analysis of log-transformed 90 bp cfDNA dynamics and laboratory parameters with 30-/180-day mortality.

|  |  | 30-day mortality | | | 180-day mortality | | |
| --- | --- | --- | --- | --- | --- | --- | --- |
|  | Parameter | **HR** | **95% CI** | ***p*-value** | **HR** | **95% CI** | ***p*-value** |
| COVID-19 sepsis | log cfDNA  (90 bp) | 1.45 | 1.12, 1.87 | 0.005* | 2.66 | 1.59, 4.45 | <0.001* |
|  | cfDNA II | 0.47 | 0.20, 1.10 | 0.08 | 0.29 | 0.05, 1.62 | 0.2 |
|  | CRP (mg/l) | 1.29 | 0.94, 1.77 | 0.1 | 1.54 | 0.92, 2.58 | 0.10 |
|  | PCT (ng/ml) | 1.30 | 1.06, 1.59 | 0.01* | 1.78 | 1.25, 2.55 | 0.001* |
|  | LDH (U/l) | 3.15 | 1.52, 6.53 | 0.002* | 12.1 | 2.87, 51.1 | <0.001* |
|  | WBC (/l) | 1.97 | 1.09, 3.58 | 0.03* | 4.61 | 1.12, 19.0 | 0.034* |
|  | Lactate (mmol/l) | 1.92 | 1.06, 3.46 | 0.03* | 4.33 | 1.60, 11.7 | 0.004* |
| Bacterial sepsis | log cfDNA  (90 bp) | 0.77 | 0.55, 1.07 | 0.12 | 1.23 | 0.72, 2.08 | 0.4 |
|  | cfDNA II | 1.29 | 0.79, 2.10 | 0.32 | 0.53 | 0.21, 1.31 | 0.2 |
|  | CRP (mg/l) | 1.18 | 0.78, 1.82 | 0.45 | 1.24 | 0.68, 2.26 | 0.5 |
|  | PCT (ng/ml) | 1.13 | 0.95, 1.34 | 0.17 | 0.93 | 0.69, 1.27 | 0.7 |
|  | LDH (U/l) | 0.33 | 0.15, 0.75 | 0.008* | 0.22 | 0.05, 0.89 | 0.034 |
|  | WBC (/l) | 1.74 | 1.01, 3.00 | 0.05* | 2.20 | 0.84, 5.78 | 0.11 |
|  | Lactate (mmol/l) | 1.35 | 0.86, 2.12 | 0.19 | 1.25 | 0.53, 2.96 | 0.6 |

Hazard ratios (HR) with 95% confidence intervals (CI) are shown; significant results (p < 0.05) are marked with an asterisk. *bp* base pairs, *cfDNA* cell free DNA, *CRP* C-reactive Protein, *II* Integrity Index, *l* liter, *LDH* Lactate dehydrogenase, *mg* milligram, *ml* milliliter, *mmol* millimoles, *ng* nanogram, * *p* < 0.05, *PCT* Procalcitonin, *U* Units, *WBC* White blood cells.
