# Supplementary material for: Prospective observational study of cell-free DNA as a prognostic biomarker in COVID-19 and bacterial sepsis: COVSEP-study
Source: Sci Rep. 2025 Dec 18;15:44144. doi: 10.1038/s41598-025-32810-4 (PMC12717081; doi:10.1038/s41598-025-32810-4)
Supplement: Supplementary file 8 — Supplementary Information 8. [file 41598_2025_32810_MOESM8_ESM.docx]

**Prospective observational study of cell-free DNA as a prognostic biomarker in COVID-19 and bacterial sepsis**

**COVSEP-Study**

Katharina Hoeter^1^, Elmo W.I. Neuberger^2^, Vanessa Jochum^1^, Robert Kuchen^3^, Kira Enders^2^, Maria Bergmann^1^, Michael K. E. Schäfer^1,4,5^, Perikles Simon^2^, Marc Bodenstein^1^

^1^Department of Anesthesiology, University Medical Centre of the Johannes Gutenberg-University, Mainz, Ger-many

^2^Department of Sports Medicine, Disease Prevention and Rehabilitation, Johannes Gutenberg-University Mainz, Mainz, Germany

^3^Institute of Medical Biostatistics, Epidemiology and Informatics, University Medical Centre of the Johannes Gutenberg-University, Mainz, Germany

^4^Focus Program Translational Neurosciences (FTN), Johannes Gutenberg-University, Mainz, Germany

^5^Research Center for Immunotherapy, University Medical Centre of the Johannes Gutenberg- University, Mainz, Germany

Corresponding author:

Katharina Hoeter, MD

katharina.hoeter@unimedizin-mainz.de

ORCID: 0000-0003-4392-9672

**Supplementary Table 5**: Correlation of log-transformed 90 bp cfDNA with inflammatory and metabolic biomarkers over time in COVID-19 and bacterial sepsis.

|  |  | **COVID-19 sepsis** | | | **Bacterial sepsis** | | |
| --- | --- | --- | --- | --- | --- | --- | --- |
| **Time point** | **Laboratory parameter** | **Cor. Coeff.** | ***p*-value** | **n** | **Cor. Coeff.** | ***p*-value** | **n** |
| **1** | LDH (U/l) | 0.79 | <0.001* | 27 | 0.61 | <0.001* | 37 |
|  | PCT (ng/ml) | -0.10 | 0.63 | 27 | 0.38 | 0.02* | 35 |
| **2** | CRP (mg/l) | 0.16 | 0.46 | 24 | 0.43 | 0.01* | 34 |
|  | LDH (U/l) | 0.72 | <0.001* | 24 | 0.58 | <0.001* | 30 |
|  | Lactate (mmol/l) | 0.25 | 0.25 | 24 | 0.41 | 0.02* | 33 |
| **3** | CRP (mg/l) | 0.54 | 0.015* | 20 | 0.14 | 0.49 | 26 |
|  | LDH (U/l) | 0.73 | <0.001* | 20 | 0.29 | 0.28 | 16 |
|  | PCT (ng/ml) | 0.57 | 0.009* | 20 | 0.09 | 0.74 | 18 |
|  | Lactate (mmol/l) | 0.15 | 0.55 | 19 | 0.49 | 0.04* | 18 |
| **4** | CRP (mg/l) | 0.83 | 0.008* | 9 | 0.33 | 0.19 | 17 |
|  | LDH (U/l) | 0.75 | 0.03* | 9 | 0.71 | 0.02* | 11 |
|  | PCT (ng/ml) | 0.98 | 0.002* | 8 | 0.66 | 0.02* | 13 |
|  | Lactate (mmol/l) | 0.43 | 0.29 | 9 | 0.6 | 0.04* | 12 |

*p-*value compares cfDNA at individual measurement time points with Laboratory parameters in Spearman’s rank correlation test, *Cor. Coeff.* Correlation Coefficient, *n* number of observations

*bp* base pairs, *cfDNA* cell-free DNA, *CI* Confidence Interval, *CRP* C-reactive Protein, *HR* Hazard-Ratio, *l* liter, *LDH* Lactate dehydrogenase, *mg* milligram, *ml* milliliter, *mmol* millimoles, *ng* nanogram, * *p* < 0.05, *PCT* Procalcitonin, *U* Units, *WBC* White blood cells.
