# Supplementary material for: Prospective observational study of cell-free DNA as a prognostic biomarker in COVID-19 and bacterial sepsis: COVSEP-study
Source: Sci Rep. 2025 Dec 18;15:44144. doi: 10.1038/s41598-025-32810-4 (PMC12717081; doi:10.1038/s41598-025-32810-4)
Supplement: Supplementary file 9 — Supplementary Information 9. [file 41598_2025_32810_MOESM9_ESM.docx]

**Prospective observational study of cell-free DNA as a prognostic biomarker in COVID-19 and bacterial sepsis**

**COVSEP-Study**

Katharina Hoeter^1^, Elmo W.I. Neuberger^2^, Vanessa Jochum^1^, Robert Kuchen^3^, Kira Enders^2^, Maria Bergmann^1^, Michael K. E. Schäfer^1,4,5^, Perikles Simon^2^, Marc Bodenstein^1^

^1^Department of Anesthesiology, University Medical Centre of the Johannes Gutenberg-University, Mainz, Ger-many

^2^Department of Sports Medicine, Disease Prevention and Rehabilitation, Johannes Gutenberg-University Mainz, Mainz, Germany

^3^Institute of Medical Biostatistics, Epidemiology and Informatics, University Medical Centre of the Johannes Gutenberg-University, Mainz, Germany

^4^Focus Program Translational Neurosciences (FTN), Johannes Gutenberg-University, Mainz, Germany

^5^Research Center for Immunotherapy, University Medical Centre of the Johannes Gutenberg- University, Mainz, Germany

Corresponding author:

Katharina Hoeter, MD

katharina.hoeter@unimedizin-mainz.de

ORCID: 0000-0003-4392-9672

**Supplementary Table 6**: Correlation of log-transformed 222 bp cfDNA with inflammatory and metabolic biomarkers over time in COVID-19 and bacterial sepsis.

|  |  | **COVID-19 sepsis** | | | **Bacterial sepsis** | | |
| --- | --- | --- | --- | --- | --- | --- | --- |
| **Timepoint** | **Laboratory parameter** | **Cor. Coeff.** | ***p*-value** | **n** | **Cor. Coeff.** | ***p*-value** | **n** |
| **1** | LDH (U/l) | 0.79 | <0.0001* | 27 | 0.53 | <0.001* | 37 |
|  | WBC (/l) | 0.5 | 0.008* | 27 | -0.15 | 0.37 | 37 |
| **2** | LDH (U/l) | 0.74 | <0.001* | 24 | 0.45 | 0.01* | 30 |
|  | IL-6 (pg/mL) | -0.21 | 0.32 | 10 | -0.78 | 0.008* | 10 |
|  | WBC(/l) | 0.3 | 0.16 | 24 | 0.38 | 0.028* | 34 |
| **3** | CRP (mg/l) | 0.48 | 0.03* | 20 | 0.17 | 0.41 | 26 |
|  | LDH (U/l) | 0.77 | <0.001* | 20 | 0.16 | 0.55 | 16 |
|  | PCT (ng/ml) | 0.65 | 0.002* | 20 | 0.42 | 0.08 | 18 |
|  | Lactate (mmol/l) | 0.24 | 0.32 | 19 | 0.67 | 0.003* | 18 |
| **4** | CRP (mg/l) | 0.78 | 0.02* | 9 | 0.47 | 0.06 | 17 |
|  | LDH (U/l) | 0.7 | 0.04* | 9 | 0.39 | 0.24 | 11 |
|  | PCT (ng/ml) | 0.93 | 0.002* | 8 | 0.76 | 0.003 | 13 |
|  | Lactate (mmol/l) | 0.37 | 0.37 | 8 | 0.71 | 0.01* | 12 |

*p-*value compares cfDNA at individual measurement time points with Laboratory parameters in Spearman’s rank correlation test, *Cor. Coeff.* Correlation Coefficient, *n* number of observations, *bp* base pairs, *cfDNA* cell free DNA, *CI* Confidence Interval, *CRP* C-reactive Protein, *HR* Hazard-Ratio, *IL-6* Interleukin 6, *l* liter, *LDH* Lactate dehydrogenase, *mg* milligram, *ml* milliliter, *mmol* millimoles, *ng* nanogram, * *p* < 0.05, *PCT* Procalcitonin, *pg* pictogram, *U* Units, *WBC* White blood cells.
