# Supplementary material for: Prospective observational study of cell-free DNA as a prognostic biomarker in COVID-19 and bacterial sepsis: COVSEP-study
Source: Sci Rep. 2025 Dec 18;15:44144. doi: 10.1038/s41598-025-32810-4 (PMC12717081; doi:10.1038/s41598-025-32810-4)
Supplement: Supplementary file 10 — Supplementary Information 10. [file 41598_2025_32810_MOESM10_ESM.docx]

**Prospective observational study of cell-free DNA as a prognostic biomarker in COVID-19 and bacterial sepsis**

**COVSEP-Study**

Katharina Hoeter^1^, Elmo W.I. Neuberger^2^, Vanessa Jochum^1^, Robert Kuchen^3^, Kira Enders^2^, Maria Bergmann^1^, Michael K. E. Schäfer^1,4,5^, Perikles Simon^2^, Marc Bodenstein^1^

^1^Department of Anesthesiology, University Medical Centre of the Johannes Gutenberg-University, Mainz, Ger-many

^2^Department of Sports Medicine, Disease Prevention and Rehabilitation, Johannes Gutenberg-University Mainz, Mainz, Germany

^3^Institute of Medical Biostatistics, Epidemiology and Informatics, University Medical Centre of the Johannes Gutenberg-University, Mainz, Germany

^4^Focus Program Translational Neurosciences (FTN), Johannes Gutenberg-University, Mainz, Germany

^5^Research Center for Immunotherapy, University Medical Centre of the Johannes Gutenberg- University, Mainz, Germany

Corresponding author:

Katharina Hoeter, MD

katharina.hoeter@unimedizin-mainz.de

ORCID: 0000-0003-4392-9672

**Supplementary Table 7:** Association of log-transformed 90 bp cfDNA levels with major clinical complications in ICU patients based on generalized estimating equations.

| **Characteristic** | **Beta** | **95% CI** | **p-value** | **R²** |
| --- | --- | --- | --- | --- |
| **AKI with RRT** | 0.202 | -0.324, 0.727 | 0.452 | 0.536 |
| **AKI w/o RRT** | 0.051 | -0.450, 0.553 | 0.841 | 0.533 |
| **ECMO** | 1.265 | 1.032, 1.499 | <0.001* | 0.559 |

*p-value* compares association of cfDNA-levels with different adverse events, *AKI* acute kidney injury*, Beta* estimated regression coefficient*, CI* confidence interval, *ECMO* extracorporeal membrane oxygenation, * *p* < 0.05, *R^2^* coefficient of determination, *RRT* renal replacement therapy.
